# Influence of Microplastics on Morphological Manifestations of Experimental Acute Colitis

**DOI:** 10.3390/toxics11090730

**Published:** 2023-08-25

**Authors:** Natalia Zolotova, Dzhuliia Dzhalilova, Ivan Tsvetkov, Olga Makarova

**Affiliations:** Department of Immunomorphology of Inflammation, Avtsyn Research Institute of Human Morphology of Federal State Budgetary Scientific Institution “Petrovsky National Research Centre of Surgery”, 117418 Moscow, Russia; natashazltv@gmail.com (N.Z.); davedm66@gmail.com (I.T.); makarov.olga2013@yandex.ru (O.M.)

**Keywords:** microplastics, polystyrene, colon, colitis, dextran sulfate sodium, mice

## Abstract

Microplastic pollution poses a threat to human health. It is possible that the increase in the incidence of inflammatory bowel disease is associated with exposure to microplastics. We investigated the effect of the consumption of polystyrene microparticles with a diameter of 5 μm at a dose of 2.3 mg/kg/day for 6 weeks on morphological changes in the colons of healthy male C57BL/6 mice and of mice with acute colitis induced by a 1% dextran sulfate sodium solution (DSS). In healthy mice, microplastics caused an increase in the number of endocrine cells, an increase in the content of highly sulfated mucins in goblet cells, an increase in the number of cells in the lamina propria, and a decrease in the volume fraction of macrophages. Microplastic consumption caused more severe acute colitis, which is characterized by a greater prevalence of ulcers and inflammation and a decrease in the content of neutral mucins in goblet cells.

## 1. Introduction

Plastic pollution is a global environmental problem. Of particular concern are small plastic particles with a diameter of less than 5 mm—microplastics (MPs). Due to the small size of the particles, MPs are intensively distributed in the environment via water and wind. MPs are found all over the globe: in the air, soil, and water, in polar ice, at the depths of the seas, and in living organisms [1,2,3]. In this regard, the question of the impact of MPs on human health is pertinent. To study the toxic effects of various substances, the pathogenesis of human diseases, and the preclinical evaluation of drugs, laboratory mice are most often used as model organisms. Therefore, in recent years, active work has been carried out to study the effects of MPs on the mouse organism.

It was demonstrated that orally consumed MPs accumulate in the large intestine of mice, causing damage to the epithelial barrier of the colon and changes in the composition of the intestinal microflora. MPs penetrate into the liver and kidneys of mice, causing inflammatory changes in these organs, a decrease in the relative weight of the liver, disturbances in carbohydrate and lipid metabolism, and oxidative stress. MPs, due to the induction of oxidative stress and damage to mitochondria, can cause the death of cardiomyocytes and the development of myocardial fibrosis. Moreover, MPs cause cognitive impairment and affect the behavior of animals, impair reproductive function, and cause disturbances in the development of offspring [1,4,5,6,7,8,9,10,11].

People consume MPs mainly via water and food, and the first target of their action is the gastrointestinal tract [3]. It is possible that the worldwide increase in the incidence of inflammatory bowel diseases (IBDs), including ulcerative colitis, is associated with an increase in the number of MP particles in the environment [12]. It was demonstrated that, in Taiwan, where the basis of people’s diet is seafood from heavily polluted waters, the incidence of ulcerative colitis over 10 years has increased by more than 1.5 times—from 0.61 per 100,000 people in 1998 to 0.98 per 100,000 in 2008 [13]. Z. Yan et al. (2022) found that the concentration of MP particles in the feces of patients with IBDs is significantly higher than that of healthy people and that there is a positive correlation between the concentration of MPs in feces and the severity of IBDs [14].

To date, only three studies on the effect of MPs on the course of experimental colitis have been published [15,16,17]. According to these studies, exposure to MPs in mice with experimental colitis causes a more pronounced shortening of the colon, increases the severity of structural damage and inflammation, reduces mucus secretion, increases colon permeability and levels of pro-inflammatory cytokines in the blood serum, and exacerbates pathological changes in the liver. However, morphological changes in the colon mucosa during colitis against the background of MP consumption have not been studied.

Therefore, the aim of this study was to evaluate the effect of microplastic consumption on morphological changes in the colon during experimental acute colitis.

## 2. Materials and Methods

The study was performed on 32 adult male C57BL/6 mice obtained from the “Stolbovaya” branch of the Federal State Budgetary Institution of Science’s “Scientific Center for Biomedical Technologies of the Federal Medical and Biological Agency”, Russia. The mice were 1.5 months old and weighed 20–24 g. The mice were kept as 8 animals per cage in an open system at a temperature of 18–21 °C with natural light and had free access to water and feed. All efforts were made to decrease suffering and possible stress for the animals; the study was performed in accordance with Directive 2010/63/EU of the European Parliament and of the Council of 22 September 2010 on the protection of animals used for scientific purposes.

**Figure 1:**
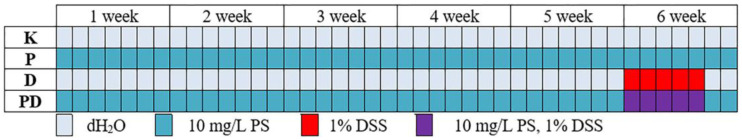
Experimental design.

The animals were divided into 4 groups of 8 mice each (Figure 1): K—the control group, in which, throughout the experiment, the mice received distilled water; P—the model of MP consumption; D—the model of acute DSS colitis; PD—the model of acute DSS colitis against the background of MP consumption. The drinking water of the animals in groups P and PD was substituted for 6 weeks with a suspension of polystyrene (PS) microparticles of 5 μm in diameter in distilled water at a concentration of 10 mg/L (79633, Sigma-Aldrich, St. Louis, MO, USA). The plastic type, particle size, and slurry concentration were consistent with previous studies [6,18]. Glass drinking vessels were used to avoid foreign plastic particles entering the water. The average weight of the mice was 22 g, the animals drank about 5 mL of the suspension per day, respectively, and the MP dose was about 2.3 mg/kg/day. Senathirajah K. et al. (2021) estimated that globally, on average, humans may ingest 0.1–5 g of MPs weekly through various exposure pathways, corresponding to approximately 0.2–10 mg/kg/day [19]. For the induction of acute colitis in the animals of groups D and PD, for 5 days (from days 36 to 40 of the experiment), dextran sulfate sodium (DSS) with a molecular weight of 40 kDa (AppliChem) was added to the drinking vessels with a final concentration of 1%. For 5 days, each animal consumed approximately 0.25 g of DSS [20]. The animals were taken out of the experiment on the 43rd day via the method of cervical dislocation under ether anesthesia.

In DSS-induced colitis, the most pronounced morphological changes are observed in the distal colon [20]. Therefore, the distal colon was taken. It was opened along the mesentery, washed with phosphate-buffered saline at pH 7.4, straightened on a filter, and fixed in 10% buffered formalin (Biovitrum, St. Petersburg, Russia) for a day. Histological sections were made with a thickness of 5 μm. The histological sections were stained with hematoxylin and eosin, alcian blue at pH 1.0, and PAS reaction and immunofluorescence staining with antibodies to macrophage marker CD68 (DF7518 Affinity Biosciences, Jhubei City, Hsinchu County, Taiwan, dilution 1/100) and endocrine cell marker chromogranin A (ab15160, Abcam Inc, Boston, MA, USA, 1/200). The fluorescently labeled secondary antibodies Goat anti-Rabbit IgG (H + L) Cross-Adsorbed Secondary Antibody, Alexa Fluor™ 488 (A11008, Invitrogen, Waltham, MA, USA, 1/300) were used. A morphological study was carried out using the program ImageJ.

To assess the prevalence of ulcers and inflammation, longitudinal sections of the distal intestine were scanned along the entire length at a magnification of 100. The total length of the section along the lamina muscularis mucosa and the length of its sections, along which ulcers and inflammatory infiltrates occurred, were measured. The percentage of the length of the intestine with ulcers and inflammation was calculated.

To evaluate inflammatory infiltration, the sections stained with hematoxylin and eosin were photographed at a magnification of 320 in 2 fields of view. We measured the area of the connective tissue of the lamina propria and counted the number of nuclei. The number of cellular elements per 1 mm^2^ of the lamina propria area was calculated.

To assess the volume fraction of macrophages, the preparations stained with antibodies to CD68 were photographed at a magnification of 200 in 2 fields of view. The images were binarized, the area with correctly oriented crypts was circled from the lamina muscularis mucosa to the lumen, and its area and the area of the macrophages were determined. The volume fraction of macrophages was calculated as the ratio of the area of CD68-positive cells to the area of the mucosa.

To assess the number of endocrine cells, the sections stained with antibodies to chromogranin A were photographed at a magnification of 100 in 2 fields of view. The area of the mucosa with correctly oriented crypts was measured. The number of chromogranin A-positive cells in the isolated area was counted. The number of chromogranin A-positive cells per 1 mm^2^ of the mucosa was calculated.

To assess the volume fraction of goblet cells and the content of highly sulfated and neutral mucins in them, the sections stained with alcian blue at pH 1.0 and with the PAS reaction were photographed at a magnification of 200 in 3 fields of view under the same lighting conditions. Binarization section images with the PAS reaction were obtained, setting the threshold so that only goblet cells were isolated. The area with correctly oriented crypts from the lamina muscularis mucosa to the lumen was outlined, and its area and the area of goblet cells were determined. The volume fraction of goblet cells was calculated as the ratio of the area of goblet cells to the area of the mucosa. On the images of the sections stained with alcian blue and after the PAS reaction, the average brightness of the goblet cell points and the background (the image area without tissue) was measured. The optical density of goblet cells was calculated as a decimal logarithm of the ratio of the average brightness of background dots to the average brightness of goblet cell dots. The higher the optical density, the higher the content of highly sulfated (alcian blue) or neutral (PAS reaction) mucins in goblet cells detected.

The obtained data were statistically processed using STATISTICA 6.0 (StatSoft, Inc., Tulsa, OK, USA). Nonparametric statistics methods were used since the samples were small (8 animals per group) and the parameter values were not normally distributed (ꭓ^2^ criterion). The samples were described in terms of the median and IQR (25%; 75%). To compare the two groups, the Mann–Whitney U-test was used, and differences were considered statistically significant at *p* < 0.05. To compare the four groups, the Kruskal–Wallis test was used; at *p* < 0.05, a posteriori pairwise comparisons were made according to the Mann–Whitney U-test with Bonferroni correction. Differences were considered statistically significant at *p* < 0.0085.

## 3. Results

**Figure 2:**
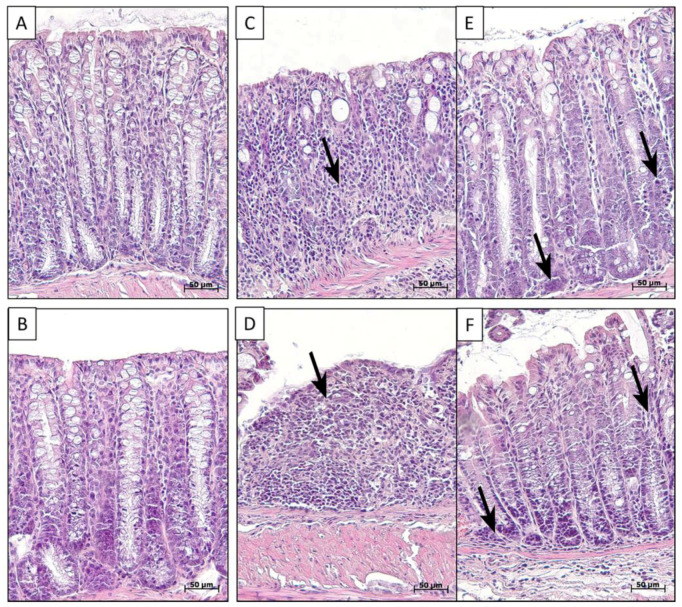
Distal colon of control mice (**A**), MP-treated mice (**B**), and mice with acute colitis that did not receive (**C**,**E**) and did receive MPs (**D**,**F**). In figures (**C**) and (**D**)—ulcers; (**E**,**F**)—inflammation (arrows). Hematoxylin and eosin staining. In control (**A**) and MP-treated (**B**) mice, mucus was normal: epi-thelium was preserved throughout the mucosa, the crypts were deep, and there were a lot of goblet cells and a small amount of immune cells. In mice with colitis which did not receive (**C**,**E**) and did receive MPs (**D**,**F**), there were acute ulcers and inflammation areas (arrows) with reduced goblet cell numbers that were infiltrated with neutrophils and lymphocytes.

After the histological study of the distal colon of control group mice (group K) and those who consumed PS particles with a diameter of 5 μm at a dose of 2.3 mg/kg/day for 6 weeks in drinking water (group P), no pronounced differences were observed (Figure 2A,B). In the distal colon of all animals, the epithelium was preserved throughout the mucosa. The crypts were deep, their openings narrow. The lamina propria and the submucosa contained a small amount of evenly distributed cellular elements—fibrocytes, fibroblasts, lymphocytes, and single histiocytes. For the animals with acute DSS-induced colitis, both the animals without MP exposure (group D) and the mice that consumed MPs (group PD), the morphological picture of the colon was mosaic. The most pronounced pathological changes in the colon were represented by extensive ulcers extending to the lamina muscularis mucosa. In areas with preserved epithelia and crypts, areas with severe inflammatory infiltration were identified. In these areas, the number of goblet cells was sharply reduced (Figure 2C–F). There were also areas that did not differ from the control group.

**Figure 3:**
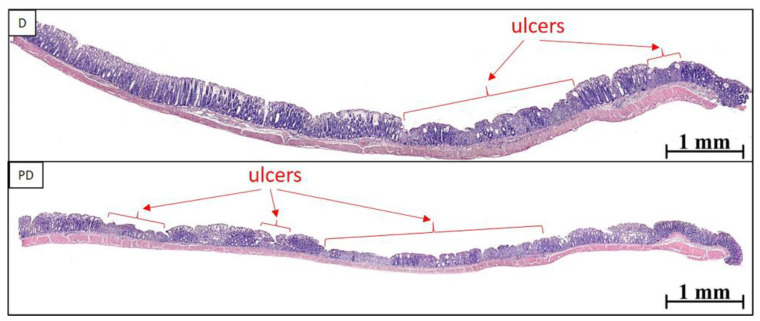
Ulcerative inflammatory changes in the distal colon of mice with acute colitis that did not receive (D) and did receive MPs (PD). Ulcers—arrows.

**Figure 4:**
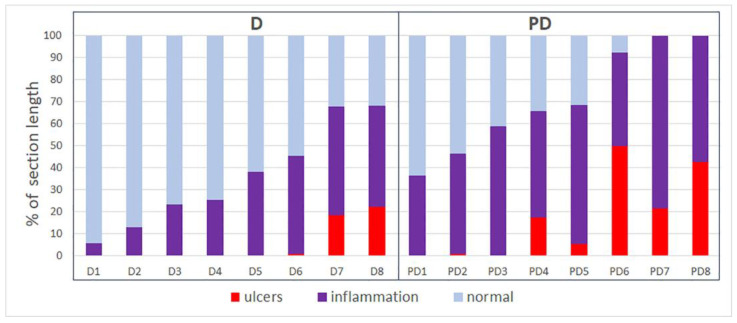
The prevalence of ulcers and inflammatory infiltration in the distal colon of mice with acute colitis that did not receive (D) and did receive MPs (PD).

**Figure 5:**
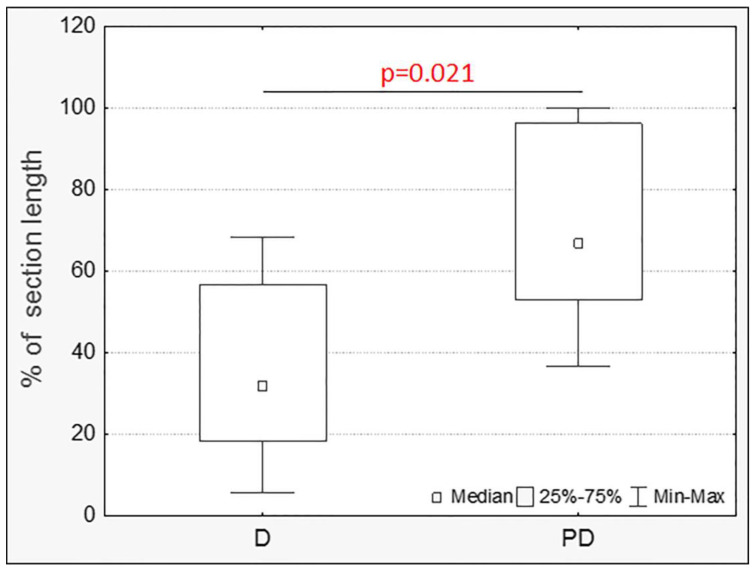
The prevalence of ulcers and inflammatory infiltration in the distal colon of mice with acute colitis that did not receive (D) and did receive MPs (PD) (Mann–Whitney test).

The prevalence of the ulcerative inflammatory process varied greatly between animals with colitis, even within the same group (Figure 3 and Figure 4); however, in the group of animals treated with MPs, the prevalence of the ulcerative inflammatory process was statistically significantly higher (Figure 5).

**Figure 6:**
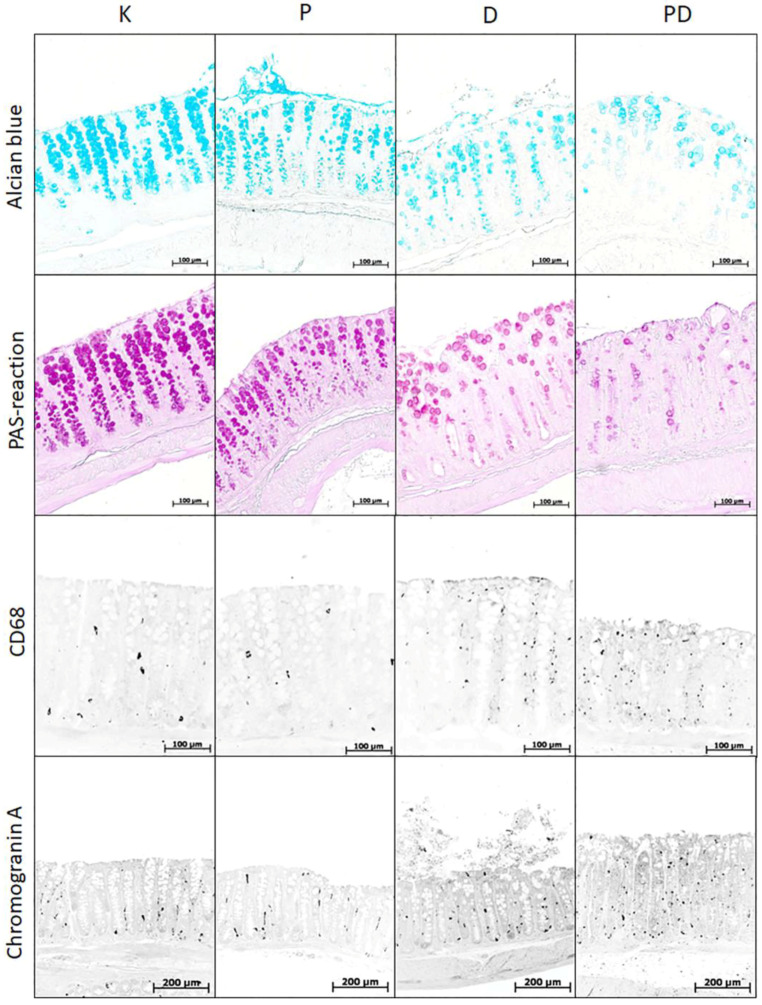
Distal colon of mice in the control group (K), consuming microplastics (P) with acute DSS-induced colitis (D) and with acute colitis against the background of microplastic consumption (PD), stained with alcian blue at pH 1.0 (highly sulfated mucins), the PAS reaction (neutral mucins), immunofluorescent staining with antibodies to CD68 (macrophages) and chromogranin A (endocrine cells). Photographs of sections with immunofluorescent staining are discolored and inverted for better contrast.

**Figure 7:**
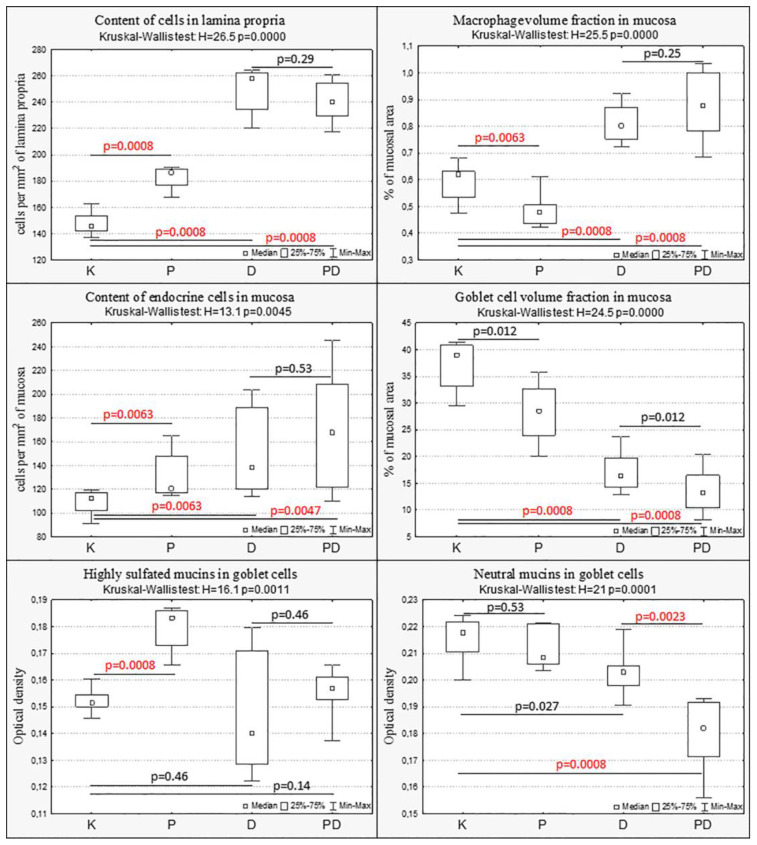
Changes in the mucosa of the distal colon with MP consumption (P), acute colitis (D), and acute colitis with MP consumption (PD) compared with the control group (K). Kruskal–Wallis test, post hoc comparisons—Mann–Whitney U-test with Bonferroni correction, statistically significant differences *p* < 0.0085.

In group P, compared with the control group, the content of cells in the lamina propria of the mucosa increased, but the volume fraction of macrophages in the mucosa decreased. The number of chromogranin A-positive endocrine cells and the content of highly sulfated mucins in goblet cells increased. There was a tendency to decrease the volume fraction of goblet cells (Figure 6 and Figure 7).

In animals with colitis, changes in the colon mucosa were assessed in areas without ulcers but with pronounced inflammatory changes. In groups D and PD, a pronounced inflammatory infiltration of the mucosa was observed, especially in the basal part of the lamina propria of the mucosa. Compared with the control group, in mice with acute colitis that consumed and did not consume MP, the content of cellular elements in the lamina propria mucosa, the volume fraction of macrophages, and the number of endocrine cells in the mucosa increased, and the volume fraction of goblet cells decreased. In the PD group, the content of neutral mucins in goblet cells also decreased (Figure 6 and Figure 7). In the PD group, compared with the D group, the content of neutral mucins in goblet cells was statistically significantly reduced (Figure 6 and Figure 7).

## 4. Discussion

### 4.1. The Effect of MPs on the Colon Mucosa in Normal Mice

To date, about 20 experimental studies using mice have been published that have evaluated the MP effect on colon structure and function. The earliest of these works came out in 2018. Dysbiosis of the intestinal microflora was revealed in mice treated with MPs [21,22,23,24,25]. According to a number of authors, exposure to MPs causes mild or moderate inflammation in the colon, characterized by weak inflammatory infiltration of the mucosa, the activation of pro-inflammatory signaling pathways, and the increased expression of pro-inflammatory cytokines [17,23,24,26,27,28,29,30]. Violations of antioxidant defense and the development of oxidative stress were revealed [28,29,30,31]. MPs cause damage to the epithelial barrier of the colon. The stimulation of apoptosis [30] and the proliferation [17] of intestinal epithelial cells were observed. A decrease in the number of goblet cells [17,26,29], mucin expression [24,31], and mucus secretion [21,22,25,30,31] were revealed. There is a decrease in the expression of tight junction protein genes [9,30] and an increase in the permeability of the intestinal barrier [28].

We found an increase in the content of cellular elements in the lamina propria of the colon mucosa in mice that consumed PS microparticles with a diameter of 5 μm at a dose of 2.3 mg/kg/day during exposure for 6 weeks, which indirectly indicates an increase in the permeability of the epithelial barrier for luminal antigens and the activation of the local compartment of the immune system. At the same time, the volume fraction of macrophages in the mucosa decreased, which was probably due to its edema. According to Li et al. (2020), C57BL/6 mice treated for 5 weeks with 600 µg/day of 10–150 µm polyethylene (PE) particles showed inflammation in the colon and a higher expression of TLR4, AP-1, and IRF5 [23]. Xie S et al. (2023) noted an increase in the levels of IL-1β and IL-6 in the colon of C57BL/6 mice fed a suspension of 5 μm PS particles at a concentration of 100 μg/L for 6 weeks [17]. Jia R et al. (2023) reported mild inflammatory infiltration of the colon, an increase in the concentration of pro-inflammatory cytokines TNF-α, IL-1β, and IL-6, and a decrease in anti-inflammatory IL-10 in C57BL/6 mice fed a suspension of polypropylene (PP) particles with a diameter of 8 microns at a concentration of 10 mg/mL for 4 weeks [30]. Rawle et al. (2022), using C57BL/6J mice treated with 1 μm PS particles at a dose of 80 μg/kg/day for 33 days via the RNA-Seq method, revealed an increase in the expression of a number of genes associated with inflammation [27]. Xie L et al. (2022) studied the effect of various types of plastic particles with a diameter of 150–300 microns on Kunming mice and found that, in animals that received 0.2 mL of a microplastic suspension at a concentration of 20 mg/mL per day for 7 days, inflammatory infiltration of the colon mucosa and its severity depended on the type of plastic: PS > PVC > PET > PE > PP [29].

We observed an increase in the content of chromogranin A-positive endocrine cells in the distal colon mucosa of mice that consumed MP. There are no published data on the effect of MPs on endocrine cells in the colon. In mice, about half of the endocrine cells in the colon are serotonin-secreting Ec-cells. [32]. Serotonin promotes mucus secretion, accelerates the release of digestive enzymes, controls the acidity of the stomach contents, slows down the absorption of water and electrolytes in the intestine, and increases its contractile activity [33]. In addition, serotonin is involved in immune responses: it stimulates the production of pro-inflammatory cytokines and the differentiation of dendritic cells, and it attracts mast cells, eosinophils, and neutrophils to the focus of inflammation [34]. It is likely that these effects of serotonin contributed to the observed increase in the number of cellular elements in the lamina propria. 

We revealed an increase in the content of highly sulfated mucins in goblet cells under the influence of MPs in the distal colon. The volume fraction of goblet cells and the content of neutral mucins in them did not change statistically significantly, although we noted a trend towards a decrease in the volume fraction of goblet cells. According to the literature, exposure to different types and sizes of MPs causes a decrease in the number of goblet cells, mucin expression, and mucus secretion [17,21,22,24,25,26,29,30,31]. There are no data in the literature on the effect of microplastics on the ratio of acidic and neutral mucins in the colon. The mucus secreted by goblet cells is involved in protecting the body from internal and external stimuli, moistening the mucosa surface, promoting hummus, and parietal digestion. It forms the outer and inner layers. The outer layer is inhabited by commensal microflora and has a loose structure, while the inner layer is dense and impervious to particles larger than 0.5 µm in diameter. A key component of mucus is the mucin Muc2, which is a highly glycosylated protein. The mucin molecule has terminal carbohydrate groups which can be either neutral or acidic. The neutral groups are not modified (-CH_2_OH) and are detected using the PAS reaction, while acidic groups are the modified residues of sulfuric (-CH_2_SO_3_-) or sialic acids and are detected using alcian blue (at pH 1.0, it stains the sulfo-groups). It is assumed that acidic mucins, especially sulfated ones, protect against bacterial translocation better than neutral ones since they are less susceptible to destruction by bacterial hydrolases [35]. Therefore, we also consider the increase in their production as a protective adaptive reaction of the organism. 

### 4.2. The Effect of MPs on the Severity of the Ulcerative Inflammatory Process and Changes in the Epithelial Barrier in Acute Colitis

There are only three studies in the literature that have evaluated the effect of MPs on the severity of acute experimental colitis. According to Zheng H. et al. (2021), in male C57 mice with acute DSS colitis treated for 28 days with a suspension of PS particles with a diameter of 5 μm, more pronounced histopathological liver damage, increased intestinal permeability, and higher levels of the pro-inflammatory cytokines IL-1β, TNF-α, and INF-γ in serum compared to mice with colitis without MPs were observed [15]. Luo T. et al. (2022) studied the effect of PS microparticles with a diameter of 5 μm on the course of acute and chronic DSS colitis. Exposure to MPs caused a more pronounced shortening of the length of the colon, exacerbated histopathological damage and inflammation, decreased mucus secretion, and increased colonic permeability. In addition, MP exposure also increased the risk of secondary liver damage [16]. Xie S. et al. (2023) induced DSS colitis in male C57BL/6 mice treated with a suspension of PS microparticles with a diameter of 5 μm for 42 days. The impact of MPs accelerated the development of colitis and led to more pronounced weight loss, diarrhea, and inflammatory changes in the colon and liver [17].

According to our data, in acute DSS-induced colitis, the prevalence of the ulcerative inflammatory process in the distal colon was statistically significantly higher in mice that consumed MP. Also, in the group of animals with colitis against the background of the consumption of MPs, the content of neutral mucins in goblet cells was lower compared to the group with colitis without MPs and the control group. Therefore, MPs lead to a more severe course of colitis. However, we did not reveal differences in the content of cellular elements in the lamina propria of the mucosa, the number of endocrine cells, the volume fraction of goblet cells, or the content of highly sulfated mucins in mice with colitis between animals treated with and not treated with MPs. Probably, the absence of differences is due to the fact that the morphological picture of the intestine was mosaic, and these parameters were not evaluated along the entire length of the section, but areas of the colon mucosa with approximately the same severity of inflammatory changes were selected. Only Xie S et al. (2023) have considered such a parameter as the number of goblet cells. The authors noted a more pronounced decrease in the number of goblet cells under the influence of MPs [17]. The remaining parameters were estimated by us for the first time.

It should be noted that in mice with colitis, the prevalence of the ulcerative inflammatory process varied greatly between animals, even within the same group. Significant variations in ulcer-inflammatory process severity between animals of one group are associated with relatively low DSS concentrations (usually, a 1.5–5% DSS solution is used for acute colitis, but we used 1%) and the individual differences in animals. We chose low concentrations of DSS to cause mild symptoms because, with severe colitis, it would have been impossible to detect the influence of MPs. Even linear animals have distinct individual differences—in the population of adult male C57BL/6 mice, it is always possible to identify 10–40% of animals with low and high resistance to hypoxia. We have previously demonstrated that, in susceptible-to-hypoxia animals, the course of acute and chronic DSS-induced colitis was much more pronounced than in tolerant-to-hypoxia mice [36,37].

### 4.3. Proposed Mechanisms of MP Action

To date, the mechanisms of the damaging action of MPs have not been studied enough. According to a review by Hirt N. and Body-Malapel M. (2020), exposure to nano- and microplastics leads to impairments of the oxidative and inflammatory intestinal balance and disruption of the gut’s epithelial permeability. Other effects of nano- and microplastic exposure include dysbiosis (changes in the gut microbiota) and immune cell toxicity. Moreover, microplastics contain additives, adsorb contaminants, and may promote the growth of bacterial pathogens on their surfaces: they are potential carriers of intestinal toxicants and pathogens that can potentially lead to further adverse effects [38].

In our work, we used sterile polypropylene spheres without additives. The particle size was 5 µm. According to Pelaseyed T. et al. (2014) [39], particles with a diameter of more than 0.5 microns cannot penetrate through the dense layer of colon mucus. Therefore, it is possible that in mice without colitis, the effects of MPs are predominantly due to changes in the microflora. In addition, we suggest that the high ratio of the surface area to the volume of MP particles can act as a sorbent and wash out the mucus layer, which reduces the protective properties of the mucus barrier and leads to an increase in its permeability. In colitis, in addition to these mechanisms, MPs can penetrate through ulcers into the intestinal mucosa and directly interact with immune cells, stimulating inflammatory reactions.

## 5. Conclusions

Polystyrene microparticles with a diameter of 5 μm at a dose of 2.3 mg/kg/day, when exposed for 6 weeks, cause changes in the colon mucosa, characterized by an increase in the number of endocrine cells in the mucosa, an increase in the content of highly sulfated goblet cell mucins, an increase in the number of cells in the lamina propria of the mucosa, and a decrease in the volume fraction of macrophages in the mucosa. The consumption of polystyrene microparticles leads to a more severe course of acute DSS-induced colitis, which is characterized by a greater prevalence of the ulcerative inflammatory process and a decrease in the content of neutral mucins in goblet cells.

## Data Availability

The data that support the findings of this study are available on request from the corresponding author.
